# Gluteal Subcutaneous Atrophy After Depot Steroid Injection for Allergic Rhinitis

**DOI:** 10.1097/WOX.0b013e3182758d80

**Published:** 2012-11-15

**Authors:** Rohan Ameratunga

**Affiliations:** 1 Clinical Immunologist, Auckland Hospital, Park Road, Grafton, Auckland 1023, New Zealand

**Keywords:** rhinitis, depot steroids

## Abstract

Allergic rhinitis is a common and often distressing condition. Currently, treatment with nonsedating antihistamines, topical therapy, and immunotherapy are very effective. Despite this, intramuscular depot steroids are commonly used in clinical practice. Here, we present the case of a young woman who developed disfiguring scarring after a depot steroid injection. This case highlights the risks of this form of treatment for allergic rhinitis.

## 

Allergic rhinitis affects up to 20% of the population. Nasal allergies cause considerable misery and are responsible for many lost days of productivity and nonattendance at school. Careful history will reveal the typical symptoms associated with allergic rhinitis. Nasal obstruction is frequently the most distressing symptom associated with poor quality of sleep [1].

Examination shows classical changes in the nasal mucosa. The seasonal nature of the symptoms will often assist with the identification of the likely triggers. Grass, weed, and tree pollens are typically seasonal but with considerable geographic variation. The tree pollen season in New Zealand, for example, is shorter than that of the northern hemisphere. These clinical impressions can be confirmed by skin prick testing or by assay of specific IgE antibodies to inhalant allergens.

Currently, most patients can be effectively managed with a combination of oral or nasal antihistamines and steroid nose sprays. In recent years, allergen-specific immunotherapy, whether given sublingually or by subcutaneous injection, has proved very effective in the management of allergic rhinitis.

Patients with unresponsive symptoms may need to be evaluated for chronic rhinosinusitis. A computed tomography and rhinoscopy may be useful in delineating nasal and sinus anatomy. Patients who fail medical therapy or those with skeletal abnormalities, such as a deviated nasal septum, may benefit from a surgical procedure such as a turbinectomy or functional endoscopic sinus surgery.

Systemic steroids have played a role in the management of allergic rhinitis in the past 5 decades. Early reports confirmed an excellent response to systemic steroids. In recent years, the use of oral steroids has not been recommended for the management of allergic rhinitis in view of the efficacy of topical therapy and concerns about adverse effects.

However, oral and intramuscular depot steroids remain popular in primary care because of their efficacy and the expense of allergen-specific immunotherapy. Here, we present the case of a patient who suffered disfiguring subcutaneous atrophy in the gluteal region from a depot triamcinolone injection given for allergic rhinitis.

## Case report

**Figure 1:**
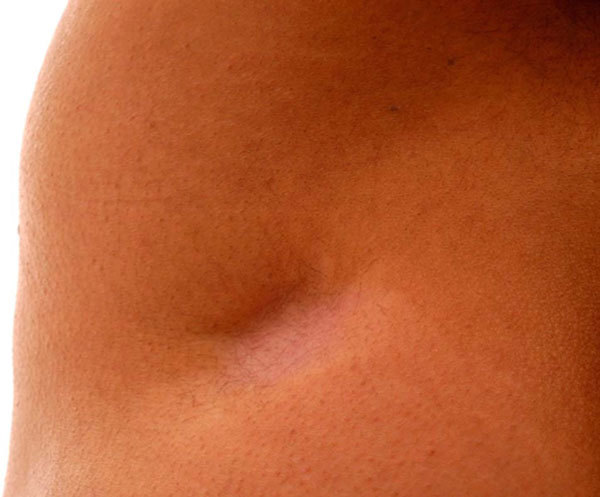
**Subcutaneous atrophy of the gluteal region after a 40-mg depot triamcinolone injection**.

**Figure 2:**
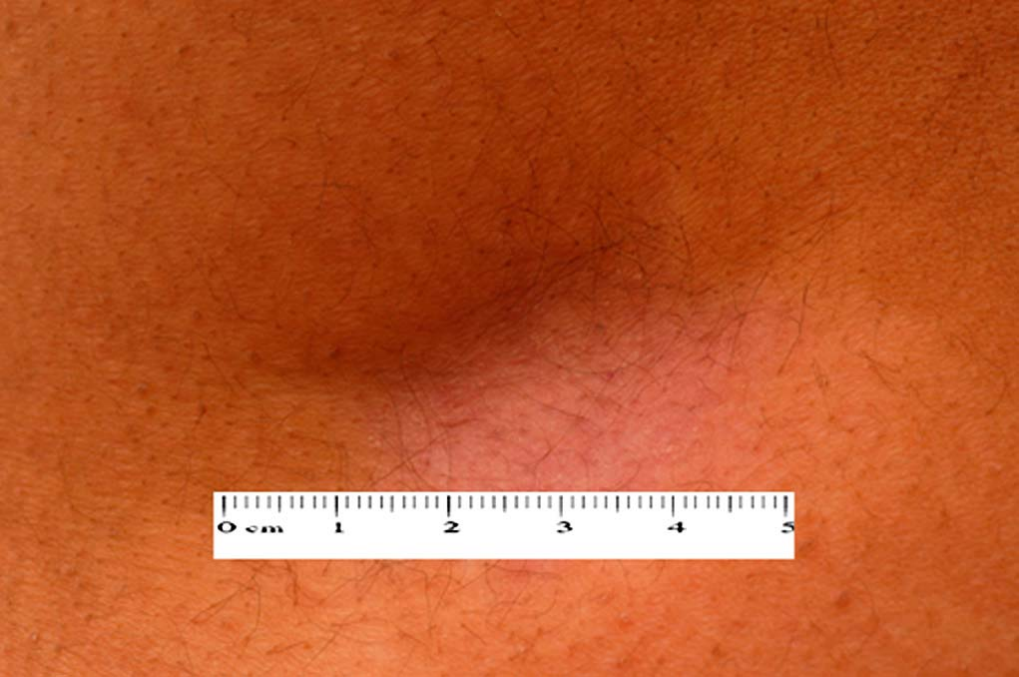
**Close-up view of the 5-cm scar after a 40-mg triamcinolone injection given for allergic rhinitis**.

The patient is a 29-year-old immigrant. Shortly after moving to New Zealand, she suffered disabling allergic rhinitis symptoms. Her symptoms were perennial. She was clinically assessed by her family physician and was given a depot 40-mg triamcinolone injection into the gluteal region. She experienced rapid relief of her nasal obstruction and rhinorrhea. She had received prior injections of depot steroids for her rhinitis. Within a few weeks, she developed a large 5-cm scar at the injection site (Figures 1 and 2).

She was reviewed in the immunology clinic at the Auckland Hospital. Skin prick testing confirmed strongly positive reactions to dust mites. She was treated with a combination of loratadine 10 mg and Budesonide 100 μg nasal spray twice daily. She declined the offer of immunotherapy on account of expense. She was reviewed by a plastics and reconstructive surgeon, but no operative intervention was offered.

## Discussion

Depot steroid injections for seasonal allergic rhinitis have been commonly used in some parts of the world, particularly Denmark. One study estimated that 0.66% of the entire Danish population received depot steroid injections each year [2].

The efficacy of depot steroid injections has been established in several randomized controlled trials [3]. Depot steroids appear to be more effective than topical beclomethasone [4] and are as effective as 7.5 mg of oral prednisone given for 3 weeks [5]. Most patients experience 4 to 5 weeks of symptoms relief after a single injection.

Generally, nasal obstruction is more likely to improve than rhinorrhea or sneezing. It is of interest that most of these randomized trials of depot steroids were undertaken in Denmark. All these randomized trials of depot steroids for allergic rhinitis were undertaken before 1988. It was around this time that effective nonsedating antihistamines became widely available.

In recent years, there has been increasing concern about the use of depot steroids in the management of allergic rhinitis [6]. Patients with severe symptoms unresponsive to antihistamines and topical steroid therapy should be referred to an allergy specialist for consideration of immunotherapy [7]. The lack of allergy services in many parts of the world, including New Zealand, has hindered such referrals [8].

Although relatively uncommon, adverse effects from depot steroids are a source of increasing concern. Bilateral avascular necrosis after a depot steroid injection has been reported [8]. There is concern about hypothalamic pituitary adrenal axis suppression and long-lasting effects on bone mineral density [6].

Subcutaneous atrophy of the deltoid has been described after depot steroid injections for allergic rhinitis. This has been termed pseudomorphea [9]. One case of subcutaneous atrophy has been estimated to occur after 11,000 injections [2]. Although this may be a rare event, there is concern about underreporting [6]. In some patients, the area of scarring improves slowly after several years.

This case illustrates the small but significant risk of using this form of treatment for a non-life-threatening disorder. Before this case, it was previously thought that intramuscular injections into the gluteal region were associated with a low risk of subcutaneous atrophy [2]. It is uncertain if this patient received an intramuscular injection or whether the triamcinolone was injected into the subcutaneous tissues.

Depot steroid injections remain very poplar among primary care physicians and patients. Their low cost and effectiveness underscore this popularity. The pressure to use this form of treatment should be resisted given the small but significant risk of disfiguring adverse effects.

A short course of oral steroids could be justified for patients attending an important function such as a wedding or before surgery in unresponsive patients. Given the availability of effective therapy, depot steroid injections should be considered an obsolete form of treatment for allergic rhinitis. This from of treatment, particularly repeated doses, should no longer be accepted as the standard of care of allergic rhinitis [10].

## Competing interests

The author declares that they have no competing interests.
